# Wastewater-Based Epidemiology for SARS-CoV-2 in Northern Italy: A Spatiotemporal Model

**DOI:** 10.3390/ijerph21060741

**Published:** 2024-06-06

**Authors:** Matilde Fondriest, Lorenzo Vaccari, Federico Aldrovandi, Laura De Lellis, Filippo Ferretti, Carmine Fiorentino, Erica Mari, Maria Grazia Mascolo, Laura Minelli, Vincenza Perlangeli, Giuseppe Bortone, Paolo Pandolfi, Annamaria Colacci, Andrea Ranzi

**Affiliations:** 1 Regional Agency for Prevention, Environment and Energy of Emilia-Romagna, 40139 Bologna, Italy; lvaccari@arpae.it (L.V.); ericamari1997@gmail.com (E.M.); mmascolo@arpae.it (M.G.M.); gbortone@arpae.it (G.B.); acolacci@arpae.it (A.C.); aranzi@arpae.it (A.R.); 2 Alma Mater Institute on Healthy Planet, Department of Biological, Geological and Environmental Sciences, University of Bologna, 40138 Bologna, Italy; federico.aldrovandi3@unibo.it; 3 Hera Tech Srl, 40127 Bologna, Italy; laura.delellis@gruppohera.it; 4 Local Health Authority of Bologna, Department of Public Health, 40124 Bologna, Italy; filippo.ferretti@ausl.bologna.it (F.F.); carmine.fiorentino@ausl.bologna.it (C.F.); vincenza.perlangeli@ausl.bologna.it (V.P.); paolo.pandolfi@ausl.bologna.it (P.P.); 5 Water Directorate, Hera SpA, 40127 Bologna, Italy; laura.minelli@gruppohera.it

**Keywords:** wastewater-based epidemiology, SARS-CoV-2, COVID-19, wastewater, biodegradation, sewer network, spatiotemporal model, public health, early-warning system

## Abstract

The study investigated the application of Wastewater-Based Epidemiology (WBE) as a tool for monitoring the SARS-CoV-2 prevalence in a city in northern Italy from October 2021 to May 2023. Based on a previously used deterministic model, this study proposed a variation to account for the population characteristics and virus biodegradation in the sewer network. The model calculated virus loads and corresponding COVID-19 cases over time in different areas of the city and was validated using healthcare data while considering viral mutations, vaccinations, and testing variability. The correlation between the predicted and reported cases was high across the three waves that occurred during the period considered, demonstrating the ability of the model to predict the relevant fluctuations in the number of cases. The population characteristics did not substantially influence the predicted and reported infection rates. Conversely, biodegradation significantly reduced the virus load reaching the wastewater treatment plant, resulting in a 30% reduction in the total virus load produced in the study area. This approach can be applied to compare the virus load values across cities with different population demographics and sewer network structures, improving the comparability of the WBE data for effective surveillance and intervention strategies.

## 1. Introduction

The global effort against the COVID-19 pandemic has underscored the need for innovative and robust surveillance methods to track the prevalence of SARS-CoV-2, the causative agent of COVID-19, within communities. Wastewater-based epidemiology (WBE) has emerged as a valuable tool for the proactive and scalable monitoring of viral presence in populations [1], and several countries have successfully implemented WBE as a surveillance system complementary to clinical testing [2,3]. This surveillance method not only aids in the early detection and prevention of disease outbreaks but also enables timely public health interventions such as targeted testing, contact tracing, and resource allocation [1,2]. Several studies [4,5] have emphasized the importance of environmental surveillance as an early warning system to detect the viral levels in the population and identify outbreaks before cases are reported to the healthcare system.

Recently, the application of this method as a complementary approach to clinical surveillance has become even more important given the reduction in recorded tests and the widespread occurrence of asymptomatic or mildly symptomatic cases [6]. Indeed, wastewater serves as a collective pool of genetic material shed by an entire community, providing insights into the prevalence of SARS-CoV-2, including asymptomatic and presymptomatic cases [1,2,7]. The advances in the monitoring of SARS-CoV-2 in wastewater have been significant, with molecular techniques such as quantitative reverse transcription—polymerase chain reaction (qRT-PCR) playing a pivotal role in the accurate quantification of viral RNA [1,3].

Italy, one of the hardest hit countries, has been monitoring urban sewage since July 2020 as part of a pilot study: the “SARI project” (Epidemiological Surveillance for SARS-CoV-2 in urban sewage in Italy), coordinated by the National Institute for Public Health and involving a network composed of regions, wastewater service providers, regional environmental protection agencies, and local health authorities [8]. 

On 17 March 2021, the European Commission recommended that EU member states begin monitoring SARS-CoV-2 in wastewater by 1 October 2021 [9]. Therefore, since October 2021, in Italy, existing research activities within the SARI project have been transformed into a surveillance system, with two main focuses: analyzing the trend of SARS-CoV-2 in urban wastewaters to predict epidemiological trends in the population and studying the spread of SARS-CoV-2 variants over time [8].

Following the principles of WBE and focusing on the surveillance objective defined by the SARI project, this study aimed to predict the temporal and spatial distribution of COVID-19 cases starting from SARS-CoV-2 detection in the main wastewater treatment plant (WWTP) of a medium-sized city in northern Italy.

This interest aligns with several studies conducted in many other countries, where various modeling techniques have been employed in the development of WBE for COVID-19 surveillance [10], including regression [5,11], artificial intelligence [12,13], and deterministic models [3,14,15,16,17].

In the present study, a deterministic model was applied: the equation upon which the model was based was initially proposed by Ahmed [14] and subsequently employed in various other studies [3,15,16,17]. The equation was adapted to integrate a spatial component, which enabled the calculation of the spatial and temporal distribution of COVID-19 cases by adjusting the SARS-CoV-2 RNA concentration data at the WWTP to take into account virus biodegradation effects.

The model accurately simulated the biodegradation of the virus along the sewer network via an approach similar to that employed by McCall et al. [18]. This estimate, together with the characteristics of the population and its geographical distribution, allowed us to determine the virus load produced in each zone within the study area and the corresponding number of new cases over time.

This approach proposes a methodology that can be effectively implemented in multiple cities where WBE is conducted. This methodology enables a reliable comparison of virus load values measured at WWTPs among cities characterized by different population demographics and sewer network structures.

The performance of the model was evaluated by comparing the estimated cases with those recorded by the regional healthcare system and considering various relevant health variables to interpret the results.

## 2. Materials and Methods

### 2.1. Study Area and Population Analysis

This study was conducted based on the measurements detected at the main WWTP in Bologna, the capital of the Emilia-Romagna region in northern Italy. The plant is located in the north of Bologna and serves the city and neighboring municipalities, catering to a capacity of 800,000 population equivalents.

The study area was defined as the area served by the WWTP. The study was conducted at the sub-municipal level; therefore, the municipalities were divided into census sections [19], which are already widely used for statistical analyses. Only Bologna was divided into statistical areas [20], as these areas are more extensive and provide more recent data than the census sections [19].

**Figure 1:**
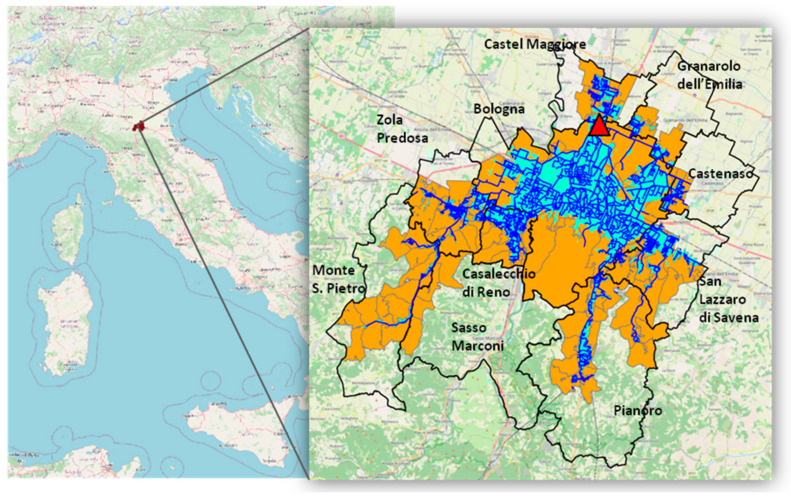
Map of the study area (orange) divided into census sections and statistical areas (delineated in gray). Representation of the wastewater treatment plant (WWTP) (red triangle), sewer network (blue), catchment area (light blue), names, and boundaries of the municipalities (black).

A three-step selection process was followed to define the areas (i.e., census sections and statistical areas) included in the study area. First, the study area was defined as the union of the areas encompassed by or intersecting the catchment area served by the sewer network. Additionally, the areas enclosed by the previously selected areas were included. Finally, those areas that were mainly connected to another treatment facility were excluded from the selected areas. The resulting study area included almost all the statistical areas of Bologna and a part of the census sections of the neighboring municipalities (Figure 1).

The study population is an estimate of the number of individuals domiciled in the study area, obtained by multiplying the number of residents [19,20] by a coefficient derived from the data in the Regional Assistance Registry, which represents the ratio of the domiciled population to the resident population at the municipal level (Table S1).

### 2.2. Virus Concentration and Wastewater Flow Rate at the WWTP

The concentration of SARS-CoV-2 RNA [GC/L] and the influent flow rate [m^3^/day] observed at the inlet of the WWTP were obtained from a data sharing platform within the SARI project network. The study period was chosen according to the availability of concentration data and ranged from 13 October 2021 (the first day of sampling) to 24 May 2023. Measurements were taken weekly or twice a week, with an irregular frequency, resulting in a total of 165 data points over the whole period.

Wastewater samples were collected and processed for RNA extraction and virus concentration determination according to the reference analytical protocol established for the SARI project [21]. Composite wastewater samples of 100 mL were collected over a 24 h period. A 50 mL aliquot was frozen and constituted the “archive sample” to be retained for any further determinations. The second 50 mL aliquot was processed immediately or stored at a refrigerated temperature (−20 °C) for a maximum of 24–48 h until analysis.

The wastewater sample concentration was achieved with a PEG/NaCl protocol according to the method published by Wu et al. [22]. The SARS-CoV-2 analysis included a first step of cell lysis with guanidine isothiocyanate, followed by extraction of the genetic material based on the adhesion of nucleic acids to magnetic silica using the MiniMag/eGeneUP (bioMerieux Italia Spa, ia di Campigliano, 58 | Loc. Ponte a Ema-50012 Bagno a Ripoli (FI), Italy) platform with a final volume of 100 µL for RNA elution. The quantitative detection of SARS-CoV-2 was performed via real-time RT-qPCR in accordance with the EU 2021/472 Recommendation [9]. The quantitative determination of SARS-CoV-2 was based on ORF-1ab (nps 14), with Murine Norovirus used as a control virus during the analysis.

The mean (1.79 × 10^5^ [GC/L]), median (0.83 × 10^5^ [GC/L]), and maximum (1.10 × 10^6^ [GC/L]) concentrations of SARS-CoV-2 RNA measured at the WWTP (Figure S2, Table S2) were within the range (10^2^–10^5^ [GC/L] with maximum values above 10^6^ [GC/L]) found in the literature [4,5,23,24].

The influent flow rate at the WWTP inlet was measured using two electromagnetic flow meters (MG3100 and MG5100) installed on two pipelines (DN 1800 and DN 1400, respectively). Typically, only one pipeline was operational at a time. However, when the flow rate increased, the second pipeline was opened and the other electromagnetic flow meter was activated. The measurements represent the average daily flow rate. In the study period, the median flow rate was 118.7 × 10^3^ [m^3^/day], the minimum flow rate was 96.8 × 10^3^ [m^3^/day], and the maximum flow rate was 432.9 × 10^3^ [m^3^/day] (Figure S3, Table S3).

In combined sewer systems, such as the one described in this study, intense rainfall events can cause the significant dilution of the SARS-CoV-2 RNA concentration in the sewer, as well as fluctuations in flow velocity and other parameters. Therefore, the concentration data collected when the flow rate exceeded the 90th percentile of the values recorded in 2022 (156.5 × 10^3^ [m^3^/day]) were treated as outliers and thus removed [11] (Table S3, Figure S3). In support of this method, the correlation between the influent flow values measured at the WWTP inlet and the cumulative daily precipitation values [25] measured by the monitoring stations in Bologna and neighboring municipalities was analyzed. The highest correlation (Pearson 0.88) was observed when considering the cumulative daily precipitation of the day preceding the flow measurement. This could be attributed to evening rains that reach the WWTP after several hours, contributing to the cumulative influent flow of the following day.

The daily load of SARS-CoV-2 RNA at the WWTP inlet [GC/day] was obtained by multiplying the observed values (after removing the outliers) of the SARS-CoV-2 RNA concentration by the influent flow rate. The historical time series of the daily SARS-CoV-2 RNA load was then processed using the locally estimated scatterplot smoothing (LOESS) method [26]. This was performed with a dual purpose: first, to transform the historical series into one with a daily regular data interval based on irregularly measured data points, and second, to smooth the values to obtain a signal purified from potential measurement errors (Figure S4). The LOESS method was implemented in Python [27], considering 11 neighboring data points for the local value estimation as in Rauch et al. [11].

### 2.3. Health Data

The Local Health Authority of Bologna provided daily COVID-19 cases within the statistical areas and census sections where the subjects were isolated.

For each patient, the following information was available: the census section or statistical area of isolation, the presence of symptoms and the date of onset of symptoms if symptomatic, the date of the swab test, diagnosis date (i.e., the date on which a positive case is officially recorded in the healthcare system), the admission date and discharge date in the case of hospitalization, the final outcome (i.e., whether the individual recovered or deceased), the final outcome date, and the cause of death if deceased. 

The model presented in this article was implemented to estimate the new daily cases. Therefore, the data on the new daily cases reported from the healthcare system were selected to evaluate the performance of the model. The decision to estimate the new daily cases instead of the active cases was made in recognition that the duration of an infected individual’s classification as an active case is likely to be more uncertain, varying significantly from person to person, and influenced by the prevailing COVID-19 regulations (e.g., a confirmatory test was not always required to confirm the end of the infection period).

To reduce the individual variability, the date of symptoms onset (or the date of the swab test if asymptomatic) was taken as the reference date for defining a new case, hospitalization, or death reported by the healthcare system. Because of the correspondence between symptoms onset and the peak of SARS-CoV-2 shedding in feces, this was considered the best reference date for comparing predicted and reported cases [15,28,29].

The daily city-level data on the tests and vaccines (second and third doses) were provided by the Local Health Authority of Bologna. An analysis of these health variables was performed to interpret the model results.

### 2.4. Model Equation

The model was based on an equation used in previous studies [3,14,15,16,17] to model the relationship between the SARS-CoV-2 concentration at WWTPs and the number of COVID-19 cases. This equation was adapted by integrating two different factors to consider the spatial component. This adjustment was made to obtain a more accurate estimate of virus biodegradation and, consequently, to derive the actual contributions of SARS-CoV-2 RNA from different areas within the catchment area and thus predict the corresponding number of COVID-19 cases.

A virus biodegradation factor and a population-based coefficient were introduced into the equation to differentiate the contribution of each area to the observed concentration at the WWTP. Both factors were calculated for each area.

The daily load of SARS-CoV-2 at the inlet of the WWTP [GC/day] was set equal to the sum of the daily virus load generated by each area, which was reduced to account for the virus biodegradation along the sewer network and refined by incorporating the population-based coefficient associated with the population of each area (Equation (1)).

(1) $$C_{W}\left(t\right)\cdot Q_{W}\left(t\right)=S_{h}\cdot M_{f}\cdot IR\left(t\right)\cdot \sum_{i}\left(P_{i}\cdot \;e^{-k{ϴ}_{i}}\cdot W_{i}\right)$$

where *C_W_* (*t*) is the measured concentration of SARS-CoV-2 RNA at the inlet of the WWTP [GC/L], *Q_W_* (*t*) is the wastewater flow rate at the inlet of the WWTP [L/day] (*C_W_* (*t*) multiplied by *Q_W_*(*t*) represents the daily load of SARS-CoV-2 RNA [GC/day] as derived in Section 2.2), *S_h_* is the fecal shedding rate (i.e., the number of SARS-CoV-2 RNA genomic copies per gram of feces produced by each infected individual) [GC/g], *M_f_* is the mass of feces produced per inhabitant per day [g/(inhabitant · day], *i* represents the area within the municipality, *P_i_* is the population in the area *i* [inhabitant], *k* is the biodegradation constant [h^−1^], *θ_i_* is the hydraulic residence time for the area *i* [h], *W_i_* is the population-based coefficient characteristic of each area *i* [adim.], and *IR*(*t*) [adim.] is the average infection rate (i.e., the number of cases divided by population) across the entire study area, and it is the unknown variable of the equation (the methodology followed to derive the equation is detailed in the Supplementary Materials, Section S3). Once *IR*(*t*) was determined, the number of cases in each area over time (*c_i_*) was derived (Equation (2)).

(2) $$c_{i}\left(t\right)=IR\left(t\right)\cdot P_{i}\cdot W_{i}$$

The model output was the number of predicted cases per day for each census section or statistical area included in the study area. However, when comparing the predicted and reported cases, the results were aggregated into grouped areas. The municipal boundaries were used to group the census sections, while for Bologna, a proximity criterion was chosen to group some statistical areas together. The aim of this approach was to increase the size of the population within each comparison zone, thereby enhancing the statistical significance when observing the spatial variability in the cases. The 88 statistical areas within the city of Bologna were therefore grouped into 59 areas, ensuring that none had less than 2000 inhabitants, while the aggregation of census sections by municipality resulted in zones with more than 2700 inhabitants (Figure S1, Table S12). 

The infection rate for each grouped area over time *IR_j_*(*t*) was then obtained (Equation (3)).

(3) $$IR_{j}\left(t\right)=\frac{c_{j}\left(t\right)}{P_{j}}=IR\left(t\right)\cdot W_{j}$$

where *c_j_* represents the number of cases in each grouped area, *P_j_* is the total population of the grouped area, and *W_j_* is the population-based coefficient for grouped areas (weighted average of *W_i_* based on the population size).

#### 2.4.1. Population-Based Coefficient

The population-based coefficient (*W_i_*) allowed us to consider for each area the following population characteristics in the prediction of cases: age, sex, family size [19,20], and comorbidities. The comorbidity data were provided by the Local Health Authority of Bologna and were obtained from different databases related to the years 2021–2022 (hospital discharge forms [SDO], territorial pharmaceutical care [AFT], direct dispensing drugs [FED], and specific pathology).

The *W_i_* coefficient, which is constant in time and variable across space, was defined as the ratio between a specific infection rate for area *i* (*A_i_*) and the infection rate for the entire study area (*A_TOT_*) (Equation (4)). To establish *A_i_* and *A_TOT_*, the Poisson regression was employed, and the regression coefficients (*β*_0_,*β*_1_,..*β_n_*) were derived from a study conducted in the municipality of Bologna from February 2020 to November 2021 [30] (Table S4). The variables (*x*_1_, *x*_2_… *x_n_*) were calculated for each area and represent the fractions of the population belonging to the following classes: age (0–21, 21–65, >65), sex (M/F), family size (1, 2, 3, >3), and comorbidities (hypertension, diabetes, and the “other comorbidities” considered in the study [30]).

(4) $$W_{i}=\frac{A_{i}}{A_{TOT}}=\frac{e^{\beta_{0}+\beta_{1}x_{1i}+\beta_{2}x_{2i}+\ldots \;\beta_{n}x_{ni}}}{e^{\beta_{0}+\beta_{1}x_{1TOT}+\beta_{2}x_{2TOT}+\ldots \;\beta_{n}x_{nTOT}}}$$

#### 2.4.2. Fecal Shedding Rate and Mass of Feces

Two average values of the fecal shedding rate (*S_h_*) were calculated using the data from the Delta and Omicron variants in six different communities in the USA [31]. The population-weighted average shedding rates for the Delta and Omicron variants included in the model were 10^8.658^ and 10^7.813^, respectively (Table S6).

Considering the evolution of the virus from the Delta variant to the Omicron variant in December 2021, the shedding rate for the Delta variant was used until 19 December, while that for the Omicron variant was applied from 3 January onwards, and a combination of the two values was used for the last two weeks of December (Table S8). The daily wet mass of feces (*M_f_*) produced by each individual was assumed to be 128 [g/(inhabitant · day)] [32].

#### 2.4.3. Biodegradation

The biodegradation of the virus along the sewer network was modeled using a first-order kinetics equation (Equation (S5)) [33,34,35]. The biodegradation constant (*k*) was calculated on the basis of the values found in the experiments [4] conducted under the conditions most similar to those of the present study. The values were adjusted to the observed temperatures in the Bologna sewer network (12 °C, 16 °C, 20 °C) [36] according to a linear equation (Equation (5)) [33,35]. The linear coefficient (*m*) and the intercept (*q*) were calculated from the literature values of *k* and temperature (*T*) [4], after which the *k* values corresponding to the observed temperatures in Bologna were determined. The three derived *k* values (0.097, 0.101, and 0.104 [h^−1^]) were included in the model, varying with the time of the year (Table S9).

(5) $$log_{10}k=m\cdot T+q$$

The hydraulic residence time (*θ_i_*) for each area was estimated by dividing the effective distance (*D_i_*), i.e., the distance calculated along the network from the centroid of each area to the WWTP, by the wastewater flow velocity along the sewer network (*v*). *D_i_* was determined by applying the Dijkstra algorithm [37] to measure the shortest path along the sewer network from the centroid of each area to the WWTP (using the GeoPandas library (version 0.13.2) in Python).

The value of *v* was assumed to be constant and equal to the value (0.8 [m/s]) used for the Milan sewer network [38] due to the similarities between the two sewer systems. This value falls within the typical range of the average velocity (0.3–0.91) in the cross section of combined sewers [39].

### 2.5. Uncertainty and Sensitivity Analysis

The model uncertainty was calculated using the error propagation formula of Li et al. [3], where the uncertainty is expressed as the relative standard deviation (RSD). The formula includes the relative standard deviations of each model parameter. These uncertainty values were derived from the literature (Section S4).

The total number of cases is included in the formula because the uncertainty associated with the shedding rate and the mass of faces of each individual decreases as the number of infected individuals increases: beyond 10 cases, the impact of these uncertainty values on the total uncertainty becomes limited [3].

The uncertainty associated with the model results varied from 0.81 RSD to 0.33 RSD depending on the total number of infected individuals in the area.

The unknown variable (*IR*(*t*)) exhibited a linear dependence on all the model parameters except *k* and *θ*. Therefore, a 1% variation in any of the parameters resulted in a corresponding variation in *IR*(*t*) of ±1%, while a 1% increase in *k* or *θ*, resulted in a corresponding increase in *IR*(*t*) of 0.35% (Equation (S9)).

## 3. Results

### 3.1. Effect of Biodegradation

**Figure 2:**
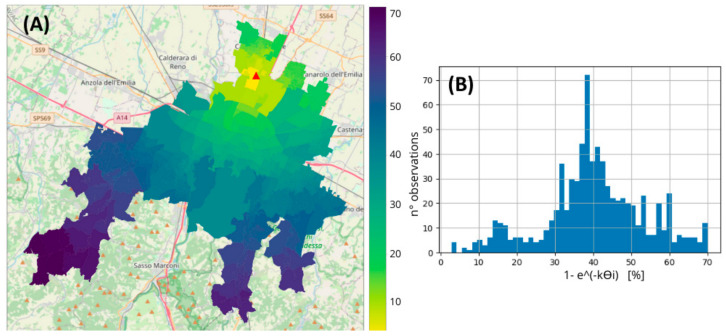
(**A**) Map of biodegradation in the study area, the WWTP (red triangle). (**B**) Frequency distribution of biodegradation in the study area. Biodegradation is expressed as a *1 − e^−kϴi^* percentage, calculated with *k* = 0.101 (*T* = 16 °C).

More than half of the areas considered were located within an effective distance range of 10 to 18 km from the WWTP (corresponding hydraulic residence time: 3.5–6 h). The virus load produced in these areas decreased by 30–45% along the path in the sewer network due to biodegradation. The virus loss was highly significant (60–70%) in the areas with the greatest effective distance (>30 km) from the WWTP (Table 1, Figure 2). However, the population density in these areas was very low.

The ratio between the SARS-CoV-2 RNA load measured at the WWTP and that generated in the areas of the catchment before entering the sewer system and thus being biodegraded is independent of time and can be expressed as (Equation (6)):

(6) $$\frac{\sum_{i}P_{i}\cdot e^{-k{ϴ}_{i}}\cdot W_{i}}{\sum_{i}P_{i}\cdot W_{i}}$$

At 16 °C, the ratio was 0.694, and this value slightly decreased as *k* increased. Therefore, the virus load detected at the WWTP was approximately 70% of that actually produced in the study area.

Starting from the estimate of biodegradation, the contribution of each area to the virus load detected at the WWTP was evaluated, and indicators were created to better understand the significance of the biodegradation and virus production in the different areas (Section S5.1).

The contribution of hospitals to the viral load discharged into the sewer system and detected at the WWTP during the study period was evaluated based on the number of hospitalized individuals. While the ratio of the COVID-19 patients hospitalized to the total number of cases reported in the whole study area was very low (0.03), this value was much higher (i.e., 42, 14, and 4) when referring only to the cases reported in the three statistical areas of Bologna where hospitals were located. These coefficients were used to estimate the virus load generated in each area with hospitals and contributing to the virus load detected at the WWTP, taking into account biodegradation. For the area where the hospital with the highest number of COVID-19 patients was located, this predicted contribution ranged from 0.014%, without considering the hospitalized subjects, to 1%, considering the hospitalized subjects (Table S13).

### 3.2. Predicted and Reported Infection Rates in the Study Area

**Figure 3:**
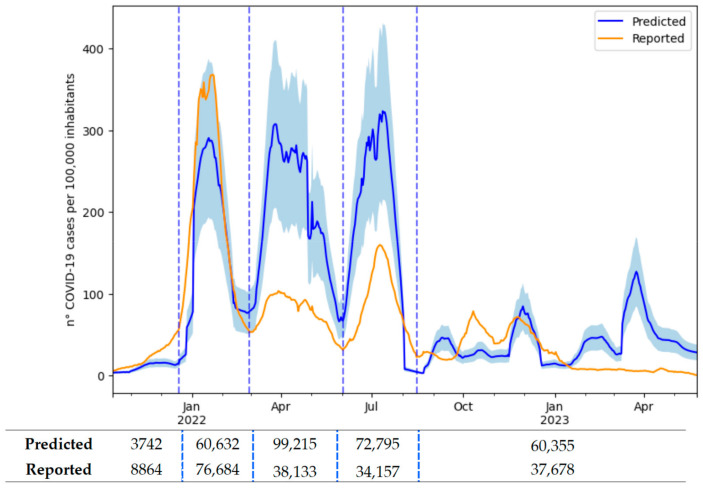
Predicted (blue) and reported (orange) infection rate *IR*(*t*) across the entire study area and the uncertainty interval (RSD) (light blue) associated with the predicted *IR*(*t*). Values of total predicted and reported COVID-19 cases over the single wave (delimitated by dashed vertical lines) in the entire study area.

The predicted and reported daily *IR*(*t*) trends over the study area were compared throughout the entire study period (Figure 3). The reported *IR*(*t*) was obtained as the daily sum of the reported COVID-19 cases in the area relative to the date of symptom onset or the date of the swab test (Section 2.3), smoothed with a simple moving average over 7 days and divided by the total population within the study area. The mean absolute error between the predicted and reported *IR*(*t*) over the entire study period was 54 cases per 100,000 inhabitants, and the Spearman correlation coefficient was 0.599. 

The time period was subdivided according to the trend of the reported cases in order to better compare the predicted and reported data. The transition dates were chosen to correspond to the relative minima of reported cases, except for the beginning of the first wave, for which the shift between the Delta and Omicron variants was chosen. This approach defined three waves and an “off-waves” period (Table 2).

The correlation was consistently high across the three waves but significantly lower during the “off-waves” period. In the first wave, the predicted and reported cases were quite similar, with an underestimation within the uncertainty range (mean absolute percentage error of 0.3), whereas in the subsequent waves, an overestimation by the model was observed (possible explanations are provided in Section 4.3).

**Figure 4:**
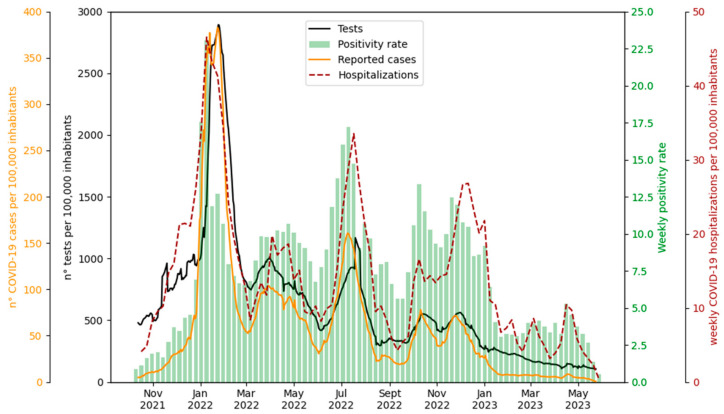
Tests: Daily reported tests across the entire study area per 100,000 inhabitants averaged with a simple moving average (SMA) over 7 days. Positivity rate: Weekly average of positive cases among tests conducted. To calculate the positivity rate, the date on which the test was conducted was taken as the reference date for both tests and cases. Reported cases: reported daily COVID-19 cases over 100,000 inhabitants averaged with a simple moving average (SMA) over 7 days (reported *IR*(*t*)). Hospitalizations: weekly sum of COVID-19 hospitalizations. Both reported cases and hospitalizations are represented based on the symptom onset date if symptomatic or the date of the test in asymptomatic cases.

The time trends for the total tests and reported cases were very similar, showing a high correlation (Spearman 0.87). For both the tests and cases, the values in the first wave were significantly higher than those observed in the subsequent waves, whereas the positivity rate (i.e., the proportion of positive cases among the tests performed) showed a less pronounced difference between the first and subsequent waves, and its trend was more similar to that of the hospitalizations (Figure 4).

In addition, to evaluate the early warning capacity of the system, the time lag between the virus load at the WWTP and the number of active cases reported over time was investigated. The diagnosis date was taken as the reference date for the active cases, corresponding to the date on which a positive case was officially registered in the healthcare system. The maximum correlation (Spearman coefficient = 0.70) was observed when comparing the virus load data with the curve of the active cases recorded 9 days later, suggesting a time lag of 9 days.

### 3.3. Spatial Comparison of the Predicted and Reported Cases in Each Grouped Area

**Figure 5:**
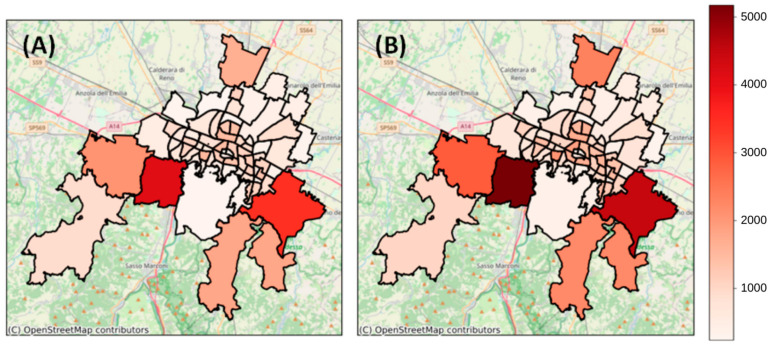
Maps of predicted total cases (**A**) and reported total cases (**B**) during the first wave in the different grouped areas.

The comparison between the predicted and reported cases in the grouped areas showed a medium/high accuracy of the model in estimating the spatial distribution of the cases (Figure 5). In particular, in the first wave, the error rate was low, with an average mean absolute percentage error across all the areas of 0.18 (min: 0.01, max: 0.20), while in subsequent waves, the error rate increased due to the greater gap between the predicted and reported daily *IR*(*t*) trends (Table S12).

**Figure 6:**
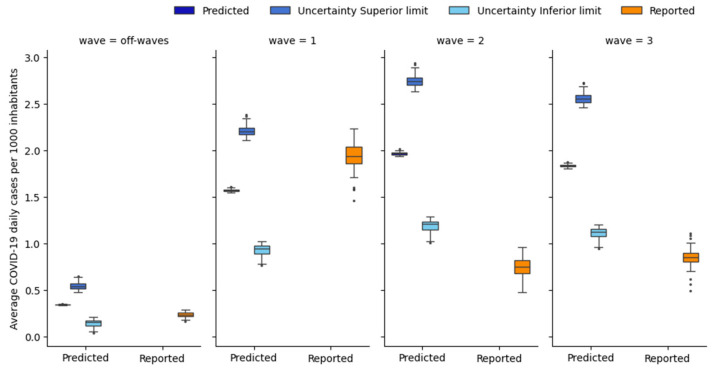
Box plot of the daily average reported and predicted infection rates (*IR_j_*), along with the lower and upper uncertainty bounds associated with the daily average predicted *IR_j_*. The *IR_j_* is expressed as daily average cases per 1000 inhabitants over the wave and in the grouped area (for each box plot, one data point for each grouped area and for each wave is represented).

However, the model failed to accurately capture the spatial variation in *IR_j_*(*t*) (Figure 6). In fact, the standard deviation of the predicted *IR_j_*(*t*) among the different areas was an order of magnitude lower than that associated with the reported *IR_j_*(*t*). The minimal variations in the predicted *IR_j_*(*t*) across the different grouped areas are due to the similarity in the values of the population-based coefficient *W_j_* (max = 1.024, min = 0.983, standard deviation = 0.009) (Table S14). 

The standard deviation of *W_j_* was low since the regression coefficients used to determine *W_j_* were very low (max = 0.329, min = −0.008) (Table S4). In addition, the population characteristics were very similar among the different areas (Table S5).

The uncertainty associated with the daily estimation of *IR_j_*(*t*) in each area was calculated (Section 2.5). As the uncertainty depends on the number of cases (the lower the number of cases, the higher the uncertainty), its value varied from area to area and was higher than that calculated in the temporal analysis. Starting from the uncertainty value, the upper and lower limits associated with the estimation of *IR_j_*(*t*) in each area were calculated (Figure 6).

Only during the first wave and off-wave periods did the reported values of *IR_j_*(*t*) remain within the uncertainty range associated with the predicted *IR_j_*(*t*), while during the second and third waves, the reported *IR_j_*(*t*) only partially overlapped with the uncertainty range.

The spatial variation in the reported *IR_j_*(*t*) did not exhibit consistency over time; across the different areas, the *IR_j_*(*t*) values deviated differently from the mean, showing both higher and lower values over time (Figure S7). Indeed, across the three different waves and in the off-waves period, only 21 out of 67 areas (31%) demonstrated consistent temporal variations. Of these, 11 areas consistently showed *IR_j_*(*t*) values above the mean, while 10 areas consistently exhibited values below the mean. However, for 46 areas, the variation was not consistent.

In a comprehensive analysis of the three waves and the off-waves, considering the variation in *IR_j_*(*t*) relative to the mean, 31 areas (46%) showed concordance between the predicted and reported variations in *IR_j_*(*t*), while this was not observed in 36 areas.

## 4. Discussion

### 4.1. Model Parameters

The fecal shedding rate (*S_h_*), the wet mass of feces (*M_f_*), and the biodegradation constant (*k*) were assumed from the literature, as the site-specific data for these parameters were not available. The shedding rate showed significant variability, ranging from 10^3^ to 10^9^ [GC/g] [3,16,29,31]. This variability was attributed to the estimation method, which can be at the individual level [3,40] or at the population level (retrospectively determined based on the observed concentrations at WWTPs and the positive cases) [16,31]. In addition, shedding depends on factors such as population characteristics (e.g., sex, age, ethnic group [31], and health status [3]), viral variants or sub-variants [31], symptoms presented, and the stage of the disease [41,42].

Given the considerable variability in shedding values, two values were selected specifically for the variants prevalent during the study period (Delta and Omicron) [31]. Moreover, we averaged the shedding values calculated across diverse and numerous populations to account for the inter-individual shedding variability.

Prasek et al. [31] considered a six-day sum of reported cases to be appropriate to represent the number of infected individuals contributing to the daily virus load in wastewater. Therefore, the shedding coefficients derived from Prasek et al. [31] were included in the model as daily averages. This allowed us to consider that the cases contributing to the concentration at the WWTP were not only the new daily cases but also the active cases with a high shedding rate, i.e., the cases occurring within the six days surrounding the day of the measurement [15,43].

The value chosen for the daily wet mass of the feces produced by each individual represents the median of data collected across the various communities in many countries over a long period (1934–2011) [32].

The value of the biodegradation constant varies depending on the type of sewer system (combined or separate), wastewater temperature and pressure [18,23,44], the initial virus concentration [18,33] and structure [34], and the other influent parameters, such as suspended solids [45], biofilm [46], BOD, and pH. The values of *k* in the literature range from 0.004 [h^−1^] [33] to 0.120 [h^−1^] [4], as determined from the experiments conducted under specific conditions. In the present study, the value for the biodegradation constant was derived from an article [4] that closely matched our conditions: the *k* value was determined by analyzing the fluctuations in the virus concentration over short time intervals, and the virus was naturally present in the collected wastewater without any additional introduction. In fact, the SARS-CoV-2 shed in feces may contain intact virus, compromised capsid virus, and free nucleic acids; thus, the rapid decay of SARS-CoV-2 RNA observed in the study [4] reflects the decay of all three viral RNA sources typically found in naturally contaminated wastewater. 

Notably, an improvement and possible future development of the model will involve the derivation of site-specific values for the described parameters.

### 4.2. Effect of the Biodegradation and Contribution of Each Area

The impact of biodegradation on reducing the viral load reaching the WWTP was assessed by estimating the reduction between the viral load generated from each area and that detected at the WWTP. The maximum reduction reached 70%, with an average reduction of approximately 30%, indicating the substantial influence of biodegradation on reducing the virus load in the sewer network.

In addition, the virus load [GC/day] produced in the entire study area over time, the removal of the effects of biodegradation, and the geographical distribution of the population were determined (Equation (6)) to allow the comparison of this parameter across different cities. According to the literature, the normalized value at the WWTP can be obtained by dividing the measured load by the total population served by the plant [47]. Our approach provides a more accurate normalization method that takes into account the actual distance of the population from the WWTP.

Furthermore, in the pursuit of the monitoring and early warning objectives, indicators were developed to assess the impact of the population density and biodegradation on the virus load detected at the WWTP. These indicators showed that densely populated areas, even those far from the WWTP, contributed significantly to the virus load at the WWTP. However, the contribution of distant areas was mitigated by the high levels of biodegradation. This led to the identification of potential monitoring sites. 

In addition, the hospital contributions to the viral load during the study period were calculated retrospectively. In fact, the contribution of each area estimated by the model is based on the population domiciled in the area and does not take into account the possibility that the infected individuals may be located in different areas. The contribution of each hospital to the total viral load at the WWTP was considered negligible, whereas the individual contribution of each hospital was significant compared to the contribution of the area in which it was located.

### 4.3. Health Variables Analysis to Compare the Predicted and Reported Infection Rates

When interpreting the results of the comparison between the reported and predicted COVID-19 cases (Figure 3), it is important to consider that the reported number of positive cases in the health surveillance system was affected by an error due to the presence of many positive individuals who were not identified. This number of unreported cases varied over the study period due to a combination of factors including the changes in the number of tests carried out and in isolation protocols, the effectiveness of the vaccine in reducing symptoms, the milder variants, and the psychological impact on individuals [6].

Identifying an appropriate time period to compare the predicted and reported cases was challenging. Ideally, validation should be performed when the number of unreported cases is as low as possible. However, the quantitative data on underreporting were not available. Therefore, a qualitative analysis of health variables that may have influenced the number of reported and predicted cases over time was conducted to better understand the comparison between them: time trends in vaccination rates, testing, and sub-variants were examined across the entire population of the study area.

From October 2021 to March 2022, the number of individuals receiving the third dose increased from 0% to 60% of the total resident population, peaking in December 2021 and January 2022. The increase in individuals receiving the third dose might have played a role in the decrease in the number of reported cases in the waves following the first wave. In fact, about 15 days after the administration of the third dose, individuals might have been less susceptible to severe outcomes, potentially affecting the detection of cases. 

The decrease in the number of tests recorded since the end of January 2022 (Figure 4) could be partly due to the introduction of rapid self-diagnostic tests in the Emilia-Romagna region from 19 January. From the positivity rate (Figure 4), it is clear that if the number of tests performed during the three waves had been equivalent, more cases would probably have been reported than were actually recorded in the second and third waves. This supports the hypothesis that the underreporting was greater in these waves. This hypothesis also seems to be supported by the hospitalization curve. In fact, the ratio of hospitalizations to new cases in January 2022 was the lowest of the entire period (2%), suggesting a greater number of unreported cases in the other waves.

**Figure 7:**
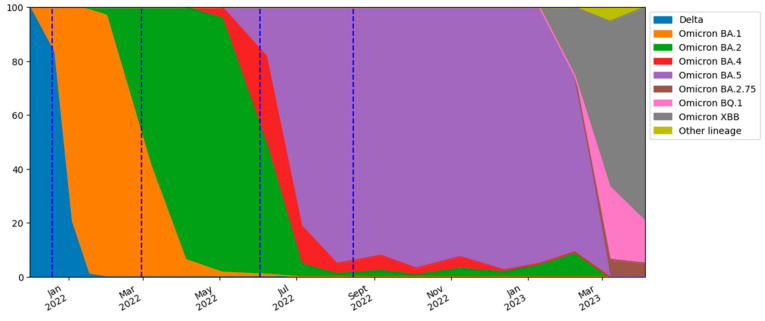
Percentage of SARS-CoV-2 variant lineages over time obtained from regional surveys.

Furthermore, a transition from the Delta variant to the Omicron variant (BA.1) was observed during the study period. According to the rapid regional surveys [48], the presence of the Omicron variant in the population increased exponentially in December 2021, overtaking the Delta variant between the penultimate week and last week of the month. This transition was followed by further variations within the Omicron lineage (BA.2, BA.4, BA.5, BA.2.75, BQ1, and XBB) (Figure 7). An increase in COVID-19 cases was observed concurrently with the variant or lineage variations (Figure 3 and Figure 7), in agreement with other studies [6,49]. Furthermore, the milder fluctuations in the predicted and reported cases in the months from October to December 2022 coincided with the minor variations in the Omicron lineages. 

It is important to emphasize that the shedding values used in the model [31] were only related to the Delta and Omicron variants (mainly BA.1). Therefore, the model overestimation in the second and third waves could also be attributed to a change in shedding due to the shift from Omicron BA.1 to BA.2 and from Omicron BA.2 to BA.4/BA.5, respectively. In fact, the shedding associated with the BA.1 sub-variant is likely to be lower than that associated with Delta and Omicron BA.2 and BA.5 [49]. However, this overestimation does not fully justify the magnitude of the observed difference between the predicted and reported cases.

In conclusion, the most reliable period for the accurate validation of the model could be from October 2021 to the end of February 2022, when the number of underreported cases was likely to be the lowest. According to the reported and predicted cases during this period, the model underestimated the number of cases reported by the healthcare system. This discrepancy, although within the uncertainty interval, is still noteworthy and may be due to the use of the parameters from the literature, such as the fecal shedding rate.

### 4.4. Spatial Comparison of the Predicted and Reported Cases in Each Grouped Area

The temporal inconsistency in the variation in the reported *IR_j_* within the same area suggests that a random effect could have caused the increase in cases in one area rather than another. Furthermore, in the areas where the variation in the reported *IR_j_* remained consistent over time, other factors such as the deprivation and obesity indices [50] may need to be considered to explain the variability in *IR_j_*. 

Additionally, a limitation of the proposed approach is the use of an estimate of the population domiciled in each area (Section 2.1) to calculate the reported *IR_j_*. In summary, the model accurately predicted the spatial distribution of cases because the reported *IR_j_* showed minimal variations among the different grouped areas. Consequently, the distribution of reported cases across the areas was primarily influenced by the population size of each area, which also determined the distribution of the predicted cases.

## 5. Conclusions

The model presented aimed to correlate the virus concentration at the WWTP with the number of COVID-19 cases in the catchment area. This approach was based on previous models that either omitted biodegradation [14,15,16,17] or treated it as a uniform factor across the catchment area [3]. Our approach included a detailed consideration of the biodegradation along the sewer system, using an approach similar to that of McCall et al. [18] but with an additional step: associating the biodegradation factor with the population distribution and characteristics.

By applying the model to the data from the main WWTP of Bologna, the daily COVID-19 cases were estimated for each census section or statistical area served by the WWTP. To evaluate the performance of the model, the predicted cases were compared with those reported by the healthcare system. The results were first analyzed by comparing the predicted and reported infection rates over time, and then by examining the variation in the different areas. 

The model performed satisfactorily in predicting the trend of reported cases over time and space. The correlation between the predicted and reported cases was consistently high across the three waves, demonstrating the model’s ability to predict the relevant waves and the significant increase in the number of cases. 

The model slightly underestimated the number of reported cases in the first COVID-19 wave and overestimated them in subsequent waves. The analysis of several health variables, in particular the tests of time trends, suggested a greater underreporting of cases by the healthcare system in the second and third waves. This could partly explain the greater difference between the predicted and reported cases in these waves and demonstrates the potential use of the model as a valuable tool for early warning systems, providing a complementary approach to clinical surveillance. In addition, a time lag of 9 days was observed between the variations in the SARS-CoV-2 load at the WWTP and changes in the trend of reported active cases.

The inclusion of biodegradation in the model equation significantly improved the case estimates and allowed the calculation of the decline in virus load along the sewer network from each area to the WWTP. Conversely, the addition of the population-based coefficient did not significantly alter the final result. 

The model estimated that biodegradation significantly reduced the virus load reaching the WWTP, resulting in a 30% reduction in the total virus load produced in the study area. The approach used in this study allows for the comparison of data from WWTPs in different cities by normalizing the detected values for virus biodegradation and the geographical distribution of the population.

Notably, potential changes to the parameters can be easily implemented to improve the accuracy of the model and can be obtained by collecting additional site-specific information and measurements (e.g., sewer flow velocity and biodegradation constant). In particular, an important future development could be the calculation of the parameter of the fecal shedding rate in situ and over time to obtain the shedding values specific to the demographic characteristics of the population and to the virus sub-variants to which the model is applied. 

In addition, the model could be optimized by placing sampling and measurement points along the sewer network. This would improve the accuracy and reliability of the case estimates for the different areas and could help to identify the local differences in infection rates. Additionally, the model can be easily integrated into an online dashboard, enhancing the accessibility and immediacy for public health officials. Finally, the model could be adapted for the surveillance of other viruses or chemicals, making it a versatile tool for monitoring and preventing the impact of different viruses or substances on the population.

## Figures and Tables

**Table 1:** Maximum, minimum, median, standard deviation, 25th percentile, and 75th percentile values of the following: linear distance of centroids from the WWTP [km], effective distance (calculated along the sewer network) of centroids from the WWTP (*Di* [km]), hydraulic residence time (*ϴ_i_* [h]), and biodegradation (*1−e^−kϴi^* [%]) varying with the biodegradation constant *k*.

	Max	Min	Median	St.Dev.	25%	75%
*Linear distance i* [km]	26.7	0.5	10.9	4.7	8.7	13.3
*D_i_* [km]	34.4	0.8	14.5	6.6	11.8	18.8
*ϴ_i_* [h]	11.9	0.3	5.0	2.3	4.1	6.5
*1 − e^−kϴi^* [%], *k = 0.097* [h^−1^]	68.6	2.8	38.6	13.0	32.8	46.9
*1 − e^−kϴi^* [%], *k = 0.101* [h^−1^]	70.0	2.9	39.8	13.2	33.8	48.3
*1 − e^−kϴi^* [%], *k = 0.104* [h^−1^]	71.1	3.0	40.7	13.4	34.6	49.3

**Table 2:** Mean absolute error (MAE), mean absolute percentage error (MAPE), and the Spearman correlation coefficient between predicted and reported *IR*(*t*) in different time intervals (first, second, and third waves, off-waves period, and the entire study period).

	1° Wave (19 December 2021–28 February 2022)	2° Wave (1 March 2022–2 June 2022)	3° Wave (3 June 2022–15 August 2022)	Off-Waves (13 October 2021–19 December 2021 and 15 August 2022–24 May 2023)	Entire Study Period (13 October 2021–24 May 2023)
MAE	47	120	109	26	54
MAPE	0.3	1.5	1.3	3.6	2.5
Spearman	0.858	0.954	0.897	-0.223	0.599

## Data Availability

Databases and information system: https://www.istat.it/en/analysis-and-products/databases (accessed on 27 May 2024); Statistical areas of Bologna: https://opendata.comune.bologna.it/explore/dataset/aree-statistiche/information/ (accessed on 27 May 2024); dext3r: https://simc.arpae.it/dext3r (accessed on 27 May 2024).

## Supplementary Materials

The following supporting information can be downloaded at: https://www.mdpi.com/article/10.3390/ijerph21060741/s1, Table S1: Resident and domiciled population in the study area; Table S2: Statistics of SARS-CoV-2 RNA concentration at the WWTP; Table S3: Statistics of wastewater flow rate at the WWTP; Table S4: Variables and estimates from the Global Poisson regression model; Table S5: Statistics of population characteristics; Table S6: Shedding rate values; Table S7: Variation of the spread of the Delta and Omicron variants and sub-variants; Table S8: Shedding values depending on the period; Table S9: Biodegradation rate constant values varying with the temperature and time of year; Table S10: Parameters in the error propagation formula; Table S11: Uncertainty associated with *IR*(*t*) varying with the number of cases; Table S12: For each grouped area: population domiciled in the area, number of reported and predicted cases, and percentage contribution of the area to the detected value of SARS-CoV-2 at the WWTP; Table S13: Contribution of hospitals in terms of viral load detected at the WWTP during the study period; Table S14: Statistics of the daily average reported and predicted infection rate and of the population-based coefficient; Figure S1: Map of areas and grouped areas included in the study; Figure S2: SARS-CoV-2 RNA concentration at the WWTP; Figure S3: Wastewater flow rate at the WWTP; Figure S4: Daily load of SARS-CoV-2 RNA at the inlet of the WWTP; Figure S5: Maps of the three indicators; Figure S6: Maps of the statistical areas of Bologna, considering hospitalized COVID-19 individuals; Figure S7: Infection rate variation across grouped areas during the study period. Equation (S1–S7): Equations used to derive the model equation; Equation (S8): Error propagation formula; Equation (S9): Normalized local sensitivity analysis; Equation (S10–S12): Equations of the three indicators; Equation (S13): Hospitals coefficient.
