# Supplementary material for: Wastewater-Based Epidemiology for SARS-CoV-2 in Northern Italy: A Spatiotemporal Model
Source: Int J Environ Res Public Health. 2024 Jun 6;21(6):741. doi: 10.3390/ijerph21060741 (PMC11203876; doi:10.3390/ijerph21060741)
Supplement: Supplementary file 1 [file ijerph-21-00741-s001.zip › ijerph-2974130-supplementary.pdf]

Supplementary Material

S1. Study area and population analysis

Table S1 Resident and domiciled population in the study area. Resident population within the study area [19,20]; percentage of residents included in the study area compared to the residents in the entire municipality; coefficient representing the ratio between domiciled and resident population at municipality level (Regional Assistance Registry); estimated number of persons domiciled in the study area

Municipality	Residents in the selected sections of the study area	Percentage of residents of the municipality included in the study area	Ratio: Domiciled/ Residents	Persons domiciled in the study area
Bologna	388588	99%	1.044	405847
Casalecchio di Reno	35745	100%	1.023	36562
Castel Maggiore	14596	79%	1.021	14908
Castenaso	2763	17%	1.022	2823
Granarolo dell'Emilia	3814	30%	1.021	3894
Monte San Pietro	7639	71%	1.007	7690
Pianoro	15739	89%	1.017	16015
San Lazzaro di Savena	30493	94%	1.005	30661
Sasso Marconi	473	3%	1.022	483
Zola Predosa	18127	95%	1.016	18410

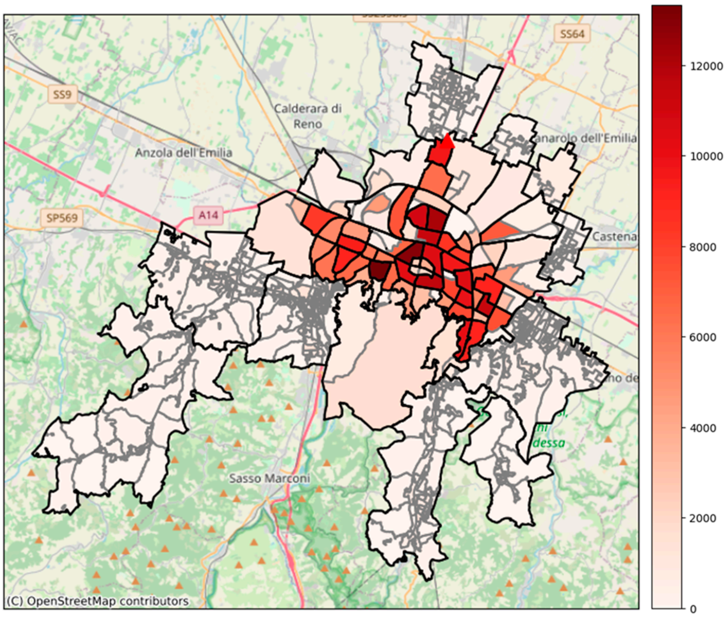

Figure S1 Map of areas and grouped areas included in the study. Grouped areas are outlined in black, and areas (census sections-statistical areas) are elineated in gray. The map's colour is based on the population domiciled in each area (census sections-statistical areas)

S2. Virus concentration (C_W), wastewater flow rate (Q_W) and virus load (M_W) at the WWTP

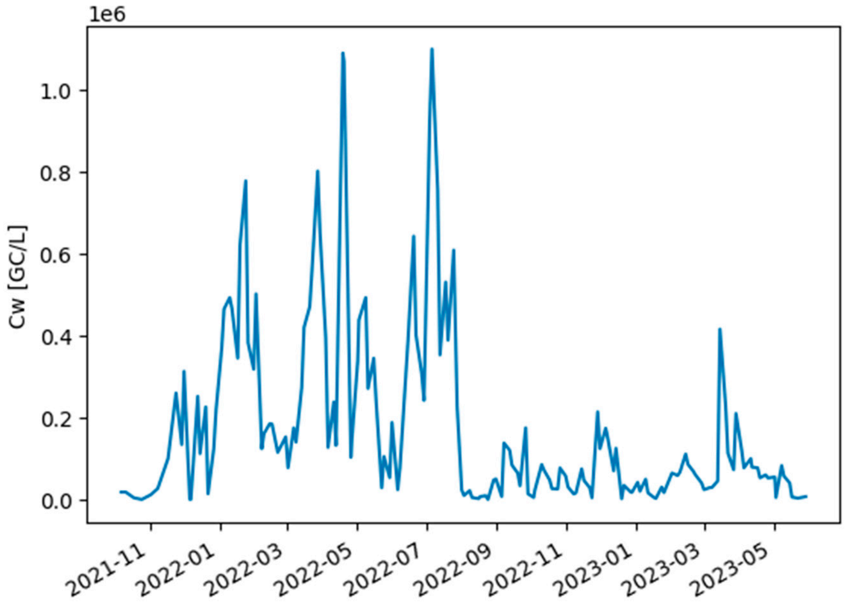

Figure S2 SARS-CoV-2 RNA concentration at the WWTP (C_W [10^6 GC/ L])

Table S2 Max., min., mean and median values of the SARS-CoV-2 RNA concentration at the WWTP (C_W [10^3 GC/ L])

	**Max.**	**Min.**	**Mean**	**Median**
**C_W [10^3 GC/L]**	1100	0	179	83

Table S3 Max., min., mean, median, percentiles values of the wastewater flow rate at the WWTP (Q_W [10^3 m^3/day])

	**Max.**	**Min.**	**Mean**	**Median**	**90º percentile**	**90º percentile (year 2022)**
**Q_W [10^3 m^3/day]**	432.9	96.8	131.0	118.7	165.6	156.5

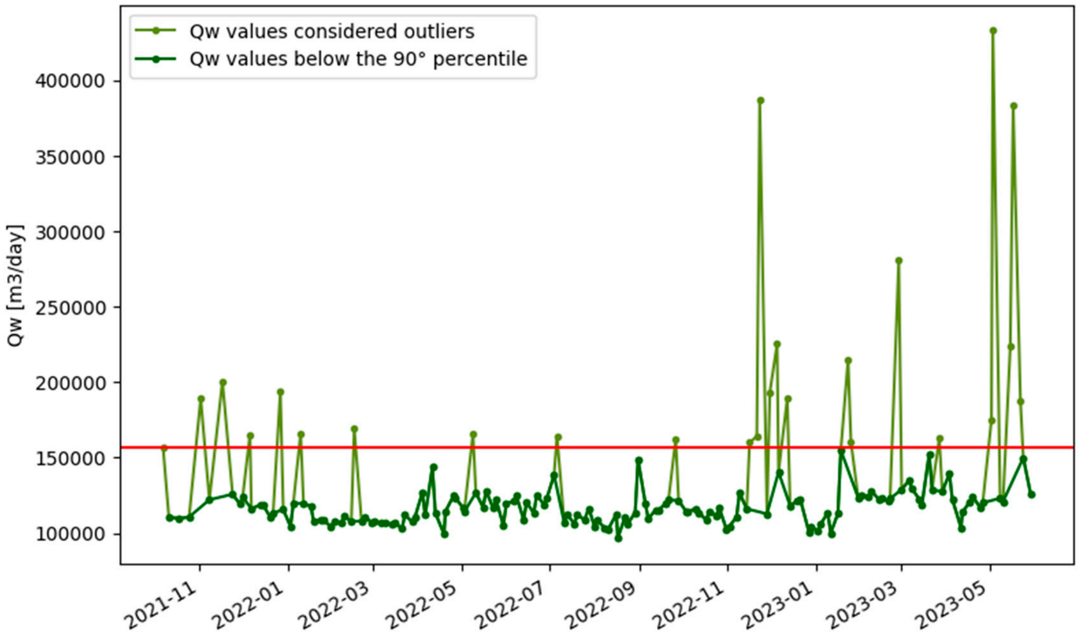

Figure S3 Wastewater flow rate at the WWTP (Q_W [m^3/day]). Red line: 90° percentile (year 2022) (156.5 10^3 m^3/day); dark green line: values below the 90° percentile; light green line: values considered outliers and thus removed from the dataset

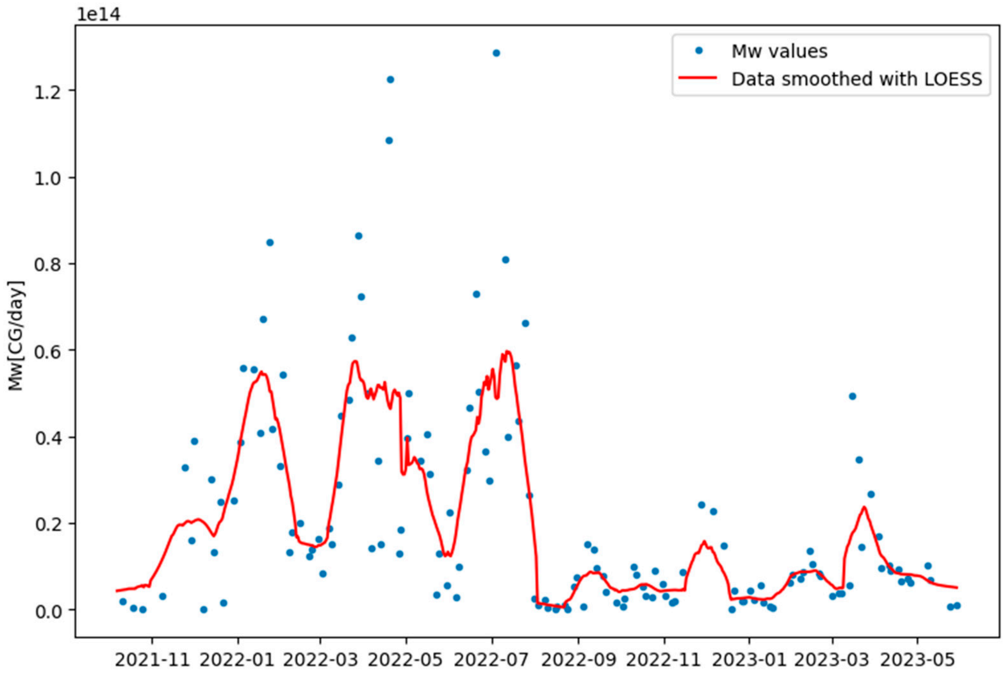

Figure S4 Daily load of SARS-CoV-2 RNA at the inlet of the WWTP (M_W [10^{14} CG/day]). In blue: values obtained by multiplying the observed values of SARS-CoV-2 concentration (C_W) by the effluent flow rate (Q_W). In red: daily trend of M_W obtained with the LOESS method

S3. Model equation

The model is based on a mass balance. The daily load of SARS-CoV-2 RNA at the inlet of the WWTP is the sum of contributions from daily load of SARS-CoV-2 RNA generated in all the selected areas. These contributions can be deduced from the population of the area using literature-based parameters related to excretion. The following equations were used to derive the model equation.

Wastewater flow rate

$$Q_W(t) = \sum_i Q_i(t) + Q_{inf}(t) \tag{S1}$$

Where:
Q_W (t) = wastewater flow rate at the inlet of the WWTP [L/day]
Q_it) = wastewater flow rates generated in the areas [L/day]
Q_{inf} (t) = total infiltration flow rate entering the sewer system [L/day]

SARS-CoV-2 RNA daily load

$$M_W(t) = \sum_i M_i(t) \tag{S2}$$

Where:
$M_W(t)$ = SARS-CoV-2 RNA daily load at the WWTP [GC/day]
M_i(t) = SARS-CoV-2 RNA daily load at the WWTP from each area [GC/day]

$$M_W(t) = C_W(t) \cdot Q_W(t) = \sum_i C_i(t) \cdot Q_i(t) \tag{S3}$$

Where:
C_W (t) is the concentration of SARS-CoV-2 RNA at the WWTP [GC/L],
C_i (t) = SARS-CoV-2 RNA concentration at the WWTP for each area [GC/L]

SARS-CoV-2 daily load generated in the i-area

$$M0_i(t) = S_h \cdot M_f \cdot c_i(t) = S_h \cdot M_f \cdot IR(t) \cdot P_i \cdot W_i \tag{S4}$$

Where:
$M0_i(t)$= SARS-CoV-2 RNA daily load produced in a single area [GC/day]
c_i (t) = infected individuals in a single area [inhabitants]
S_h = fecal shedding rate: number of SARS-CoV-2 RNA gene copies per gram of feces [GC/g]
M_f = mass of feces produced per inhabitant per day [g/(inhabitant day]
W_i = population-based coefficient [adim.]
P_i = population in the i-area [inhabitants]

Biodegradation

The model accounts for virus biodegradation along the sewer network by representing this phenomenon with a first-order kinetics. Considering each flow rate (Q_i) independently, for each area, a degradation of the SARS-CoV-2 RNA concentration occurs as follows:

$$C0_i \cdot e^{-k\Theta_i} = C_i \tag{S5}$$

Where:
$C0_i$= SARS-CoV-2 concentration in each area [GC/L]

Assuming that each Q_i is independent, it is also reasonable to assume that the Q_i remains constant throughout the sewer network. Hence:

$$\mathrm{C0_i} \cdot \mathrm{Q_i} \cdot \mathrm{e}^{-\mathrm{k\Theta_i}} = \mathrm{C_i} \cdot \mathrm{Q_i} \tag{S6}$$

This leads to:

$$M0_i(t) \cdot \mathrm{e}^{-\mathrm{k\Theta_i}} = M_i(t) \tag{S7}$$

The final model is obtained by summing these terms over all areas. Thus, we obtained, on one side, the daily load of SARS-CoV-2 RNA at the inlet of the WWTP, and on the other side, the sum of the daily loads of SARS-CoV-2 RNA generated in each area of the study area, each reduced by its biodegradation in the sewer.
Contribution of SARS-CoV-2 RNA load from each area
Compared with the SARS-CoV-2 concentration, the contribution of SARS-CoV-2 RNA load proves to be a more significant parameter for investigating each area's contribution to the observed SARS-CoV-2 RNA concentration at the WWTP. In this context, the concentration refers to the daily load of SARS-CoV-2 divided by the volumetric flow rate of wastewater. Because both the virus daily load and wastewater flow rate are strongly dependent on the population count, the ratio between the two values, (i.e. SARS-CoV-2 RNA concentration), should not vary significantly from area to area. On the other hand, the daily viral load provides direct information about the extent of each area's contribution before and after virus biodegradation along the sewer network.

Infiltration along the sewer network
The concentration data at the WWTP already account for the dilution caused by the infiltration along the sewer network, as the flow rate at the inlet of the WWTP is the sum of the flow rates generated in each area plus the infiltration flow rate. Therefore, there is no need to include any infiltration term in the model equation.

Table S4 Variables and estimates from the Global Poisson regression model [30]

Variable	Estimate (β)
Male	0.014
Age [0-21)	-0.008
Age [21-65)	- 0.068
Family size -1	-0.007
Family size -2	- 0.015
Family size -3	0.024
Hypertension	0.243
Diabetes	0.329
Any co-morbidity	0.139

Table S5 Statistics of population characteristics in the grouped areas. (expressed in fractions of population)

	Age [0-21)	Age [21-65)	Male	Family size -1	Family size -2	Family size -3	Diabetes	Hypertension	Any co-morbidity
**mean**	0.196	0.557	0.477	0.497	0.253	0.233	0.063	0.002	0.171
**max**	0.278	0.625	0.534	0.667	0.324	0.355	0.103	0.003	0.225
**min**	0.154	0.469	0.447	0.338	0.182	0.146	0.028	0.001	0.116
**std**	0.024	0.040	0.015	0.082	0.036	0.048	0.013	0.001	0.020
**median**	0.193	0.566	0.476	0.478	0.252	0.239	0.063	0.002	0.170
**25° percentile**	0.181	0.532	0.466	0.438	0.223	0.190	0.054	0.001	0.159
**75° percentile**	0.208	0.582	0.486	0.563	0.279	0.267	0.072	0.002	0.180

Table S6 Shedding rate (S_h) values. The values refer to the Delta variant and Omicron variant calculated across different communities [31], population-weighted mean and standard deviation of the values

Community	Population served	Median Delta (log_{10})	Median Omicron (log_{10})
A	18000	7.77	7.51
B	34778	7.91	7.88
C	735869	8.03	7.75
D	85712	8.13	7.77
E	401301	9.09	7.92
F	24895	8.17	
**TOTALE**	1300555		
**Population-weighted mean**		**8.658**	**7.813**
**Standard deviation**		**0.38**	**0.34**

Table S7 Variation of the spread of Delta and Omicron variants and subvariants in the Italian and regional population during the study [48]

Period	Source	Delta [%]	Omicron BA.1 [%]	Omicron BA.2 [%]	Omicron BA.4 [%]	Omicron BA.5 [%]	Other lineage
25/09/21 -8/11/21	National-level survey: integrated surveillance system	91.2					
9/10/21-22/11/21	National-level survey: integrated surveillance system	91.4					
23/10/21 –6/12/21	National-level survey: integrated surveillance system	86.6					
20/12/21	Regional-level rapid survey	83.5	16.5				
3/01/22	Regional-level rapid survey	20.6	79.4				
17/01/22	Regional-level rapid survey	1	98	1			
31/01/22	Regional-level rapid survey		97	3			
7 /03/22	Regional-level rapid survey		41.7	58.3			

04/04/22	Regional-level rapid survey		6.4	93.6			
03/05/22	Regional-level rapid survey		1.72	94.25	4.03		
07/06/22	Regional-level rapid survey		1	48.5	32.3	18.2	
05/07/22	Regional-level rapid survey			4.8	14.2	81	
02/08/22	Regional-level rapid survey			1.3	3.7	95	
06/09/22	Regional-level rapid survey			2.4	5.6	92	
04/10/22	Regional-level rapid survey			0.9	2.4	96.7	
08/11/22	Regional-level rapid survey			3.2	4.3	92.5	
13/12/22	Regional-level rapid survey			1.9	0.7	97.4	
10/01/23	Regional-level rapid survey			5		95	
07/02/23	Regional-level rapid survey			9.2		65.5	25.3
07/03/23	Regional-level rapid survey						100(XBB:61.3, BQ.1: 26.9)
04/04/23	Regional-level rapid survey						100(XBB:79.3, BQ.1: 15.6)

Table S8 Shedding values applied in the model depending on the period

Period	Until 19 Dec 2021	20 - 26 Dec 2021	27 Dec 2021 - 2 Gen 2022	After 3 Gen 2022
S_h	$10^{8.658}$	$0.75 \cdot 10^{8.658} + 0.25 \cdot 10^{7.813}$	$0.25 \cdot 10^{8.658} + 0.75 \cdot 10^{7.813}$	$10^{7.813}$

Table S9 Biodegradation rate constant (k) values applied in the model varying with temperature (T) and time of year (month)

**Months**	**T [°C]**	**k [h^{-1}]**	**k [day^{-1}]**
Dec, Jan, Feb	12	0.097	2.33
Mar, Apr, May, Sept, Oct, Nov	16	0.101	2.41
Jun, Jul, Aug	20	0.104	2.51

S4. Uncertainty and Sensitivity Analysis

To calculate the model uncertainty, the Li et al. [3] error propagation formula was used: Eq. (S8)

$$U_{IR} = \sqrt{U_S^2 + U_A^2 + U_{QW}^2 + U_k^2 + U_\theta^2 + U_P^2 + \frac{U_{Sh}^2 + U_{Mf}^2}{n}} \qquad \text{(S8)}$$

Table S10 Parameters applied in the error propagation formula (Eq. S8). Definitions, values and description of the value estimation. (The uncertainty related to W_i has been disregarded, as the coefficient has very similar values among different areas and close to 1 and has minimal impact on the calculation of IR(t))

**Parameter**	**Parameter definition**	**Value [RSD]**	**Estimation of parameters uncertainty**
U_{IR}	uncertainty related to the estimation of the infection rate (IR)		
U_S	uncertainty associated with the sampling of SARS-CoV-2	5%	assumed from the literature for the composite sampling [3]
U_A	uncertainty associated with the analysis of SARS-CoV-2 in the sample	26.5%	average between the max. and min. values found in the literature for the

			analysis of SARS-CoV-2. [3]
U_{QW}	uncertainty associated with the wastewater flow rate (Q_W)	1%	A low value was chosen for the uncertainty associated with the flow measurement at WWTP since in the study were used observed values, not design flow values
U_k	uncertainty associated with the biodegradation rate constant (k)	10.5%	was calculated based on the mean and standard deviation of measurements of the k constant in wastewater at a temperature of 15°C [23]
U_Θ	uncertainty associated with the hydraulic residence time (Θ)	4%	[18]
U_P	uncertainty related to the estimation of the population served by the WWTP	16%	Even though the estimation of the domiciled in each area used in the model is fairly accurate, there remains an error associated with the inability to control people's movements (tourists, commuters, events) [3]
U_{Sh}	uncertainty associated with the shedding rate (S_h)	$3.4 \cdot 10^{-8}$	was calculated considering the RSD of the parameter S_h used in the model **[**31**]**. A very low value was found as there is a certain level of uncertainty, but it becomes negligible compared to the parameter value
U_{Mf}	uncertainty associated with the mass of feces produced per inhabitant per day (M_f)	74%	was calculated considering the RSD of the parameter Mf used in the model (Rose et al. 2015).
n(t)	number of cases in the catchment area served by the WWTP	-	They vary daily and represent the output of the model, so every day, depending on the number of cases, the uncertainty associated with this estimate is calculated

Table S11 Uncertainty associated with IR(t) expressed as relative standard deviation (RSD) varying with the number of cases (n)

U_{IR}, n= 1	U_{IR}, n= 100	U_{IR}, n= 1000
0.81	0.34	0.33

A normalized local sensitivity analysis was conducted on the model using Eq. (S9) to examine the influence of each parameter on the value of the final result.

$$SC = \frac{\frac{A_{+1\%} - A}{A}}{\frac{P_{+1\%} - P}{P}} \quad \text{(S9)}$$

Where: SC represents the sensitivity coefficient calculated for each parameter, $P_{+1\%}$ and P represent the value of the parameter increased by 1% and the original value, respectively. $A_{+1\%}$ and A are the areas under the IR curve for the reference period, the first obtained by increasing the parameter value by 1% ($P_{+1\%}$), and the second obtained with the original parameter value (P).

S5. Results

S5.1. Indicators - Contribution of each area to the SARS-CoV-2 load at the WWTP - assessment of the risk of undetectable outbreaks

In pursuit of monitoring and early warning goals, indicators were created to better understand the significance of biodegradation and virus production in different areas. These indicators are independent of time, and for simplicity, they have been constructed disregarding the coefficient Wi, which would lead to only a minimal variation in the indicator.

To evaluate the contribution of each area to the virus load measured at the WWTP, two indicators were created (I_{DB} and I_{PB}). The I_{DB} is defined as the product of population density and the biodegradation term and was calculated for each area:

$$I_{DB} = D_i \cdot e^{-k\theta_i} \quad \text{(S10)}$$

The I_{DB} indicator showed that high-density areas were found to be very impactful, including those far from the WWTP, however, for the latter the high biodegradation value significantly reduced their contribution.

The I_{PB} represents how much each grouped area (portion of a municipality or aggregated statistical areas) contributes percentagewise to the measured SARS-CoV-2 load at the treatment plant. In other words, given a measured value at the treatment plant, this indicator represents the contributions from each area. The I_{PB} is defined as:

$$I_{PB} = \frac{\sum_{i=1}^{n} P_{ij} \cdot e^{-k\theta_{ij}}}{\sum_{i=1}^{m} P_i \cdot e^{-k\theta_i}} \quad \text{(S11)}$$

Where j indicates the grouped areas, n is the total number of areas in the grouping and m is the total number of areas in the study area.

The greater the population and population density of the area and its proximity to the WWTP are, the greater the contribution to the introduction of the virus load into the plant, and indicator (I_{DB} and I_{PB}) values increase.

Furthermore, another indicator (R_{DB}) was created following the purpose of identify areas where a possible outbreak would be difficult to detect promptly observing the concentration at the treatment plant, and therefore where sampling would be advisable. R_{DB} was defined as the product of population density and the inverse of the biodegradation term.

$$\mathrm{R}_{DB} = D_i \cdot e^{k\theta_i} \quad \text{(S12)}$$

This indicator aimed at highlighting densely populated areas that would provide a significantly contribute to SARS-CoV-2 mass production but would be challenging to detect due to the high biodegradation resulting from the considerable distance of these areas from the WWTP.

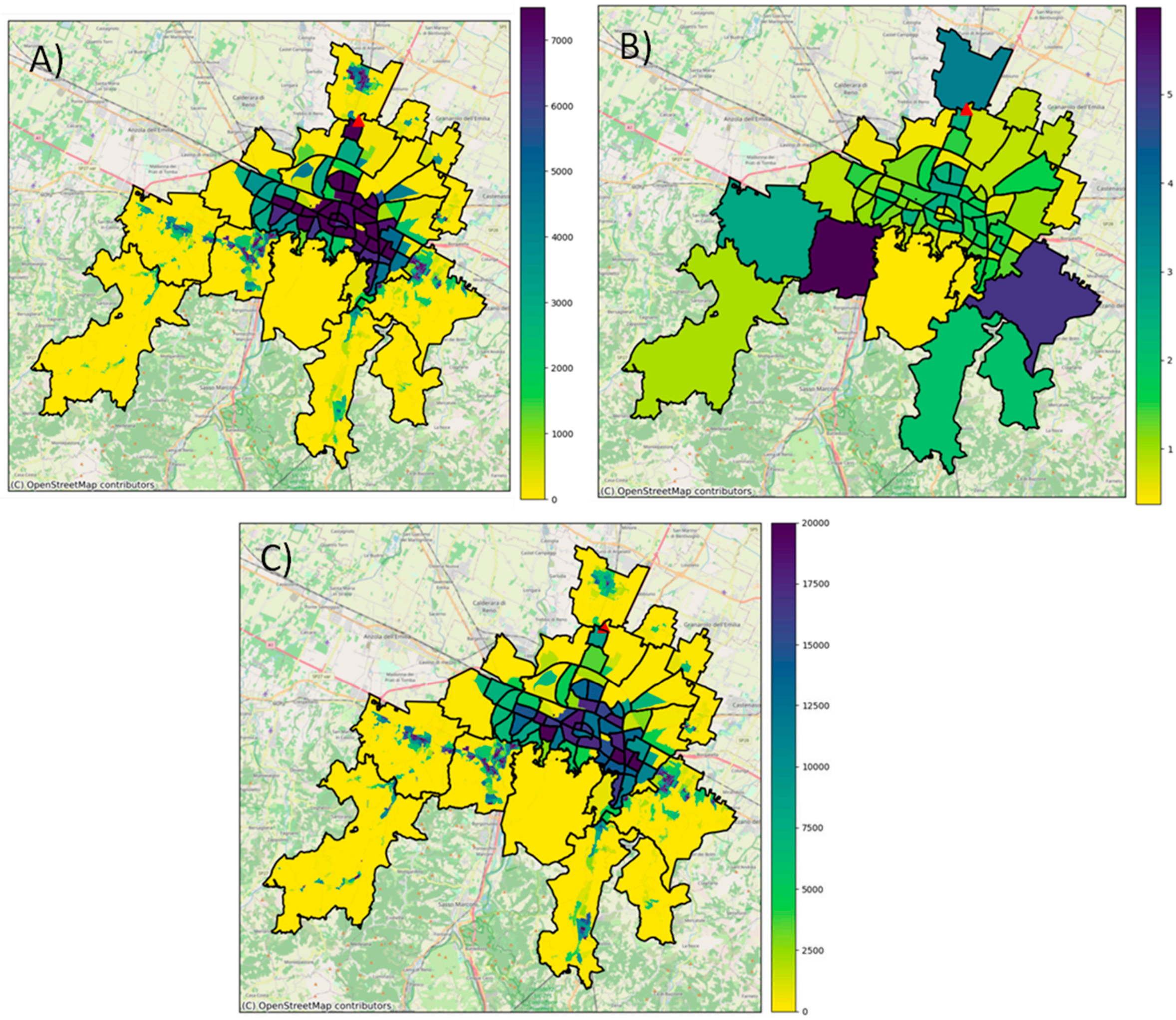

Figure S5 Maps of the 3 indicators, considering the biodegradation constant at 16°C (k=0.101 h^{-1}). A) $\boldsymbol{I_{DB}}$ indicator calculated for each area. B) $\boldsymbol{I_{PB}}$ indicator represented as a percentage, calculated for each grouped area. C) $\mathbf{R_{DB}}$ indicator calculated for each area.

Table S12 For each grouped area: population domiciled in the area, number of reported and predicted cases (during each wave) and percentage contribution of the area to the detected value of SARS-CoV-2 at the WWTP ($\mathbf{I_{PB}}$)

		**Cases off-waves**		**Cases wave 1**		**Cases wave 2**		**Cases wave 3**		
**Grouped areas**	**population**	**reported**	**predicted**	**reported**	**predicted**	**reported**	**predicted**	**reported**	**predicted**	$\boldsymbol{I_{PB}}$
Casalecchio di Reno	36562	3153	4347	5171	4114	2455	6731	2249	4940	6%
San Lazzaro di Savena	30661	2525	3659	4490	3462	2211	5665	1893	4158	5%
Zola Predosa	18410	1783	2191	2903	2073	1293	3393	1171	2490	2%
Pianoro	16015	1388	1915	2235	1812	1056	2965	970	2176	2%
Castel Maggiore	14908	1436	1765	2348	1670	926	2733	958	2006	3%
XXI Aprile	13343	1181	1584	2051	1499	995	2453	826	1801	3%
Marconi-2	12939	1160	1530	1870	1448	1053	2369	961	1739	3%
Ex Mercato Ortofrutticolo-Piazza dell'Unita'	12815	920	1524	1686	1443	807	2361	750	1733	3%

Via Ferrarese	12197	947	1445	1579	1367	795	2238	782	1642	3%
Galvani-2	11667	900	1376	1633	1302	810	2131	634	1564	2%
Bitone	11332	945	1353	1442	1280	745	2095	775	1538	2%
Arcoveggio	10872	931	1296	1482	1226	720	2007	673	1473	2%
Irnerio-2	10464	826	1227	1453	1161	809	1900	643	1395	2%
Mengoli	10432	970	1239	1568	1173	928	1919	716	1408	2%
Malpighi-2	10321	917	1217	1638	1151	840	1884	707	1383	2%
Via Toscana	10109	819	1202	1398	1138	770	1861	553	1366	2%
Dagnini	9951	895	1188	1313	1124	754	1839	603	1350	2%
San Savino	9643	778	1166	1346	1103	587	1805	649	1325	3%
Cirenaica	9555	749	1142	1379	1080	749	1768	660	1298	2%
Emilia Ponente	9411	786	1122	1435	1061	658	1737	613	1275	2%
Corelli	9168	720	1110	1221	1050	713	1718	581	1261	2%
Fossolo	9141	905	1108	1320	1049	761	1716	595	1259	2%
Battindarno	9093	823	1096	1228	1037	624	1698	556	1246	2%
Via del Lavoro	8414	596	1005	962	951	535	1556	543	1142	2%
Via Mondo	8221	758	983	1105	930	536	1522	583	1117	2%
Borgo Centro	8189	654	981	1100	929	402	1520	512	1115	2%
Monte San Pietro	8174	624	969	1033	917	506	1501	485	1101	1%
Ospedale Sant'Orsola-Mezzofanti	7935	632	945	1095	894	648	1463	443	1074	2%
Chiesanuova	7703	655	914	1012	865	628	1415	483	1039	1%
Pilastro-Scalo Merci San Donato-CAAB	7691	482	939	874	889	389	1455	419	1068	1%
Ospedale Bellaria-Via Arno	7659	709	929	1058	879	591	1439	537	1056	1%
Scalo Ravone-Prati di Caprara-Ospedale Maggiore-Zanardi	7342	623	872	1035	825	603	1350	472	991	2%
Beverara	7311	620	883	977	836	511	1368	455	1004	2%
Guelfa	7252	602	863	1099	817	566	1336	471	981	1%
San Giuseppe	6581	534	783	875	741	507	1213	428	890	1%
Canale di Reno	6575	572	789	810	747	478	1223	470	897	1%
Croce Coperta	6351	583	762	904	721	422	1180	423	866	2%
Villaggio della Barca	6236	438	755	866	715	333	1170	365	858	1%

Velodromo	6044	535	720	945	681	512	1115	446	818	1%
Croce del Biacco-Stradelli Guelfi-Roveri	5793	385	690	747	653	348	1068	307	784	1%
Lazzaretto-Lungo Reno-Tiro A Segno-Pescarola	5689	492	685	858	648	389	1061	417	779	1%
Via Vittorio Veneto	5634	491	668	781	632	487	1034	453	759	1%
Pontevecchio	5505	525	652	790	617	394	1010	326	741	1%
Triumvirato-Pietra	5413	407	648	757	613	315	1003	354	736	1%
Casteldebole	5305	414	644	667	610	337	997	371	732	1%
Ravone-Stadio-Meloncello	5094	389	604	726	572	327	936	285	687	1%
Michelino	4736	393	564	692	534	352	873	307	641	1%
Siepelunga	4683	365	556	709	527	351	862	273	632	1%
Ducati-Villaggio Ina-Rigosa	4614	437	559	705	529	282	866	296	635	1%
Agucchi	4576	326	546	631	517	283	845	281	620	1%
Osservanza	4519	346	535	663	507	351	829	206	608	1%
Cadriano-Calamosco-Fiera-San Donnino	3914	370	470	568	445	323	728	305	535	1%
Granarolo dell'Emilia	3894	287	457	538	432	252	708	203	519	1%
Irnerio-1	3848	262	452	475	428	294	700	234	514	1%
Malpighi-1-Galvani-1	3817	308	450	515	426	283	697	260	512	1%
Cavedone	3278	269	396	497	375	233	613	182	450	1%
Lungo Savena-Due Madonne	3228	247	389	443	369	225	603	214	443	1%
La Dozza-Mulino del Gomito-Savena	3226	297	386	467	365	290	598	264	439	1%
San Michele in Bosco-Giardini Margherita	3132	175	368	360	349	140	571	114	419	1%
Scandellara-Via Larga	3082	305	369	422	349	240	571	191	419	1%
Monte Donato-Ponte Savena-La Bastia	2902	268	347	439	328	216	537	173	394	0%
Castenaso	2823	218	331	297	313	141	513	167	376	1%
Bargellino-Aeroporto-La Birra	2609	193	311	357	295	175	482	167	354	0%
Marconi-1	2456	182	289	381	274	185	448	143	329	0%
Paderno-San Luca-Via del Genio	2416	156	288	356	272	138	445	101	327	0%
CNR-Caserme Rosse	2317	193	278	329	263	162	430	152	316	1%
La Noce-Laghetti del Rosario	2102	149	251	337	238	124	389	110	285	0%

S5.2. Impact of the presence of hospitals in the area

For each statistical area where a hospital is located, the coefficient to consider the presence of the hospital in the area has been defined as the ratio of COVID-19 patients hospitalized in the statistical area to the number of positive cases reported among residents in the area:

$$Hospitals\ coefficient = \frac{Total\ \text{COVID} - 19\ \text{patients in the hospital located in the i} - \text{area}}{Total\ \text{COVID} - 19\ \text{cases among residents in the i} - \text{area}} \tag{S13}$$

The coefficient was calculated considering the total number of cases and hospitalizations over the entire study period: 13/10/2021-24/05/2023. The trend over time of new daily hospitalized individuals has been similar to that of new daily cases. Therefore, the ratio of hospitalized individuals to cases has not varied significantly over time.

Table S13 Contribution of hospitals in terms of viral load detected at the WWTP during the study period based on the number of hospitalized individuals

Hospitals	Estimate of COVID-19 hospitalized patients in each hospital	Total cases reported among residents in the hospital's area	Hospitals coefficient	Total cases estimated by the model among residents in the hospital's area	Viral load contributed to the WWTP from the area (considering hospitalized individuals) as a percentage of the total viral load detected at the WWTP
Sant'Orsola Hospital	2962	70	42	43	1%
Maggiore Hospital	2584	181	14	274	0.9%
Bellaria Hospital	280	63	4	72	0.1%
Total	5826	314		389	

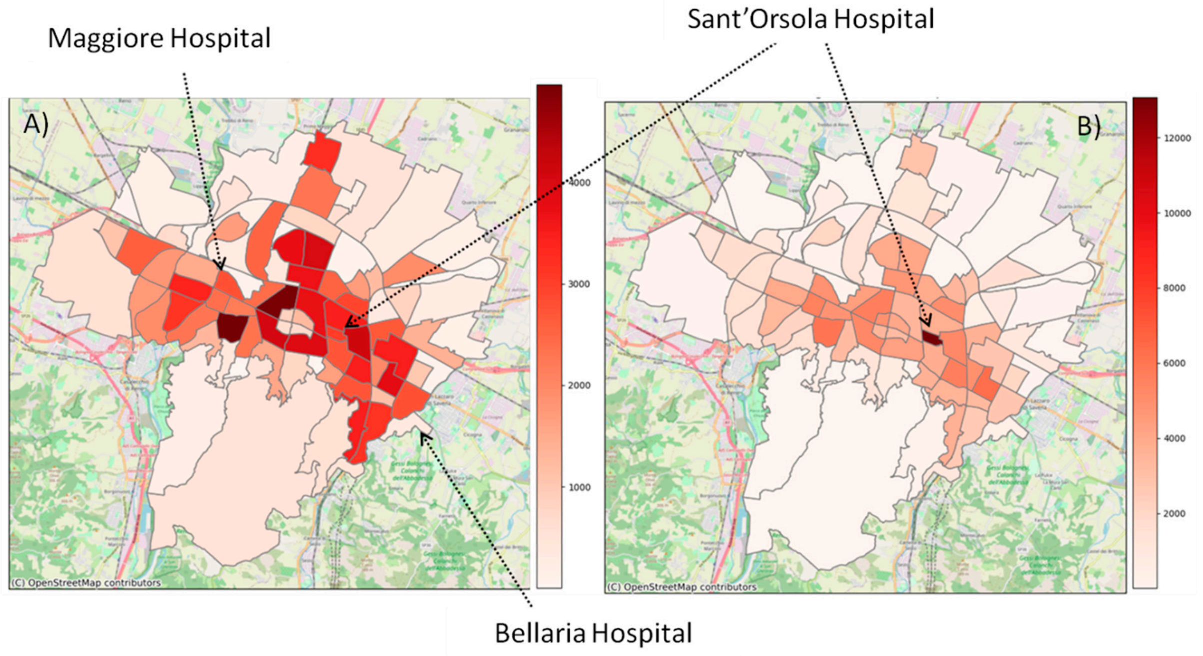

Figure S6 Maps of the statistical areas of Bologna, considering hospitalized individuals with COVID-19 within hospital areas over the entire period. A) Geographic distribution of reported cases. B) Density map of reported cases (number of cases divided by the area of the statistical area)

S5.3. Spatial comparison of estimated and reported cases in each area

Table S14 Max, min, median and st.dev. of the daily average reported and predicted infection rate (IRj, expressed as daily average cases per 1000 inhabitants over the wave and in the grouped area). Max, min, median and st.dev values of the population-based coefficient calculated for the grouped areas (W_j)

	IR_j reported 1° wave	IR_j reported 2° wave	IR_j reported 3° wave	IR_j reported off-waves	IR_j predicted 1° wave	IR_j predicted 2° wave	IR_j predicted 3° wave	IR_j predicted off-waves	W_j
max	2.23	0.96	1.10	0.28	1.60	2.01	1.87	0.35	1.024
min	1.46	0.47	0.49	0.16	1.54	1.93	1.80	0.34	0.983
median	1.94	0.74	0.85	0.24	1.57	1.97	1.83	0.34	0.999
Std. Dev.	0.16	0.10	0.11	0.03	0.014	0.018	0.017	0.003	0.009

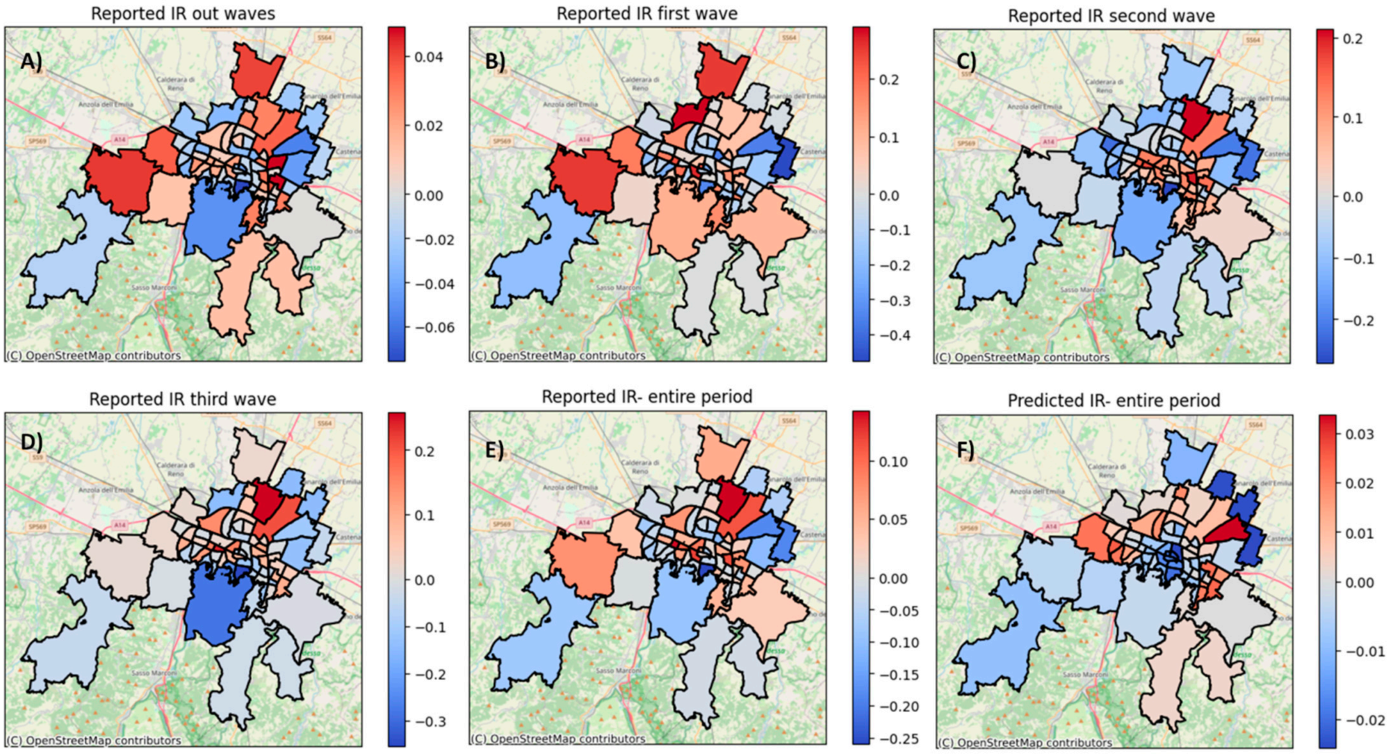

Figure S7 Infection rate variation across grouped areas during the study period. A), B), C), D), E): differences between the daily average reported infection rate in each area (IRj(t)) and the daily average reported infection rate averaged over the entire basin (IR(t)) for each wave and in the entire study period. F) Difference between the daily average predicted infection rate (IRj(t)) in each area and the daily average predicted infection rate averaged over the entire basin (IR(t)) for the entire study period. The IR is expressed as cases per 1,000 inhabitants (daily average over the period considered)
